# The effect of obesity on treatment outcomes for low back pain

**DOI:** 10.1186/s12998-016-0129-4

**Published:** 2016-12-12

**Authors:** Stanley C. Ewald, Eric L. Hurwitz, Anupama Kizhakkeveettil

**Affiliations:** 1 University of Western States, 2900 NE 132nd Avenue, Portland, OR 97230 USA; 2 Office of Public Health Studies, University of Hawaii, 1960 East-west Road, Honolulu, HI 96822 USA; 3 Southern California University of Health Sciences, 16200 Amber Valley Drive, Whittier, CA 90604 USA

**Keywords:** Low back pain, Body mass index, Obesity, Disability, Prognosis

## Abstract

**Background:**

The objective of this study was to estimate the effect of obesity, as measured by body mass index (BMI), on treatment outcomes for low back pain (LBP).

**Methods:**

Data from the University of California, Los Angeles, and Friendly Hills Healthcare Network low back pain study (collected from 1995 to 2000) were used to perform a secondary data analysis of this randomized clinical trial on adults who sought care for LBP. BMI was the primary predictor variable. Binary logistic regression modeling was performed to estimate odds ratios adjusted for the effects of confounders.

**Results:**

Using normal weight as the referent population, underweight and overweight populations did not display significant odds ratios for any of the outcome variables. The obese population demonstrated odds ratios of 0.615 (0.379, 0.998) for improvement of disability and 0.550 (0.341, 0.889) for improvement of most severe back pain.

**Conclusion:**

The results of this study support an association between obesity and less effective treatment outcomes whether measured by disability (Roland-Morris scale) or pain (most severe pain NRS). Overweight and underweight populations do not appear to have significantly different outcomes than normal weight populations.

**Trial registration:**

This trial was designed and conducted prior to the advent of registries.

## Background

It is estimated that 30 % of the global population suffers from LBP and 80 % experience LBP at some point in their lives [1, 2]. In the United States, low back pain (LBP) is one of the most common medical burdens to cause loss of work time and disability [2, 3]. LBP is considered the most common work-related disability and second most common neurological ailment [2]. Additionally, LBP accounts for heavy economic, societal, and human burden [4].

A number of epidemiological investigations have been performed to determine the work-related risk factors that lead to LBP. It has been found that occupational factors such as prolonged sitting and standing, awkward lifting, and kneeling highly contribute to LBP [5, 14]. Even though genetics also play a role, research showed that individuals with LBP often engage in tedious jobs that require lifting objects or sitting and standing for long periods of time [6].

Age is a risk factor because the chances of experiencing LBP increases as one gets older [7, 8]. One in four persons over 80 years old experiences LBP, with people aged 41–50 years old experiencing LBP (28.5 %) within a 1 month period of time [7]. Muscle elasticity and bone strength decrease as people age, resulting in the loss of flexibility and fluidity in the disc reducing the ability to protect the vertebrae [9]. Literature indicates the abuse of drugs, tobacco, and alcohol also increases the risk for LBP [10]. Many individuals who suffer from LBP also smoke cigarettes and consume alcohol [10, 11].

Race is an additional risk factor for LBP [12]. According to Waterman, Belmont, & Schoenfeld, African Americans and Caucasians are more likely to have LPB than Asians [13]. This LBP incidence report based on racial background was consistent with the findings of Knox et al., who found that among military personnel, African Americans have the highest incidence of LBP at 43.7 for every 1000 people and Asians have the lowest incidence of LBP at 30.7 for every 1000 people [12].

Obesity is another common risk factor for LBP [14, 15]. Research demonstrated that obese people treated for LBP will experience better outcomes when they lose weight, particularly in cases of morbid obesity where the body mass index (BMI) is 40 and above [16]. An enlarged abdomen as a result of obesity has been shown to cause early degeneration of discs, which is associated with LBP [17]. Obesity is associated with disc degeneration because increases in body weight lead to tear and wear on discs and joints, increasing the physical demands on muscles and ligaments [17].

There are numerous health hazards associated with obesity, including stroke, heart disease, hypertension, cancer, diabetes, gallstones and gall bladder disease, gout, osteoarthritis and problems in sleeping [18, 19]. Therefore, it is always prudent to recommend weight loss to obese patients. Nonetheless, the role of obesity as a cause, as well as its reversibility as a cure for LBP remains unclear. Even though obesity has been found to be associated with LBP, it is not clear if obesity is a cause or a consequence of LBP [5, 14]. This study examined the relationship between BMI and LBP treatment outcome.

## Methods

### Data sources

This study used data from the UCLA Low Back Pain Study conducted from 1995 to 2000. In this study, 681 participants with LBP were enrolled. Comprehensive data were collected regarding the current episode of LBP, as well as the LBP history of each participant [20]. Supplementary information was collected regarding the demographics, occupation history, disability, health status and mental health status of each patient. Outcomes were collected on each patient at 2 weeks, 4 weeks, 6 weeks and 6 months after the initial enrollment. This study used the data gathered at the 6-month follow-up period. There was a 4.3 % loss to follow-up, leaving 652 participants.

### Study design

Patients presenting to the Friendly Hills Healthcare Clinic with a complaint of LBP were offered information regarding the study. Those interested and agreeing to participate were subsequently enrolled. Individuals presenting with fractures, tumors, infections, rheumatic disease or other severe coexisting conditions were excluded from participation in the study. The majority of clinic patients belonged to a HMO and did not have to pay for services. Patients utilizing MediCal or Medicare insurance, had a Workman’s Compensation injury, or who were fee for service or had services paid by a third-party payer were eliminated from consideration. Also eliminated were patients who were unable to effectively communicate in English and those who had been under care for LBP within the previous 1 month. Of the 1203 potential study participants, 273 (22.7 %) were excluded due to ineligibility and 249 (20.7 %) were eligible but declined participation.

Patients who were enrolled were randomized into one of four treatment groups: medical care only, medical care with physical therapy, chiropractic care only, or chiropractic care with the use of physical modalities. Participants were then treated according to the treatment plan prescribed by their assigned doctor.

### Outcome variables

Participants completed the Roland-Morris disability questionnaire at baseline and at the 6-month follow-up period. The difference between these two scores was measured, and an improvement of three or more was considered a positive outcome (clinically meaningful improvement). Participants also completed an 11-point numerical rating scale (NRS) in which 0 is no pain and 10 is unbearable pain, indicating the average and most severe level of LBP they had experienced in the previous week. Again, the 6-month value was subtracted from the baseline value. Clinically meaningful improvement was considered to be a positive change on the NRS greater than or equal to two.

### Exposure variable

BMI was used as the primary exposure variable. It was modeled two ways. The first was according to National Health Guidelines, in which BMI less than 19.0 is considered underweight, greater than or equal to 19.0 and less than 25.0 is considered normal weight, greater than 25.0 and less than 30.0 is considered overweight, and greater than 30.0 is considered obese [21, 22]. BMI was also considered in a dichotomous format, obese (>30) versus not obese (≤30). BMI data was available for 618 study patients for which 6 month follow up occurred. Of these, 7 were categorized as underweight (1.13 %), 178 as normal weight (28.80 %), 250 as overweight (40.45 %), and 183 obese (29.61 %).

### Potential confounders

Potential confounders were sociodemographic variables (gender, race, age), lifestyle variables (smoking status, alcohol consumption, coffee consumption, weight change over 6 months), LBP variables (duration of current episode, history of previous episode, baseline Roland-Morris and NRS scores), assigned treatment group and patient confidence in the treatment plan. Race was categorized as white and non-white. Age was categorized by decade. Due to the low number of participants under the age of 20 and over the age of 79, participants under 20 were combined with 20–29 year olds to create a single category. Participants 80–95 were similarly combined into a single category. Other than weight change, lifestyle variables were all set as dichotomous (yes/no), as was the history of LBP variable. Duration of LBP was dichotomized to less than 3 months vs. over 3 months. Weight change was categorized as lost weight, gained weight, or had little or no weight change (less than five pounds in either direction).

Confidence was categorized as high (8–10 on a 0–10 scale), moderate (4–7) and low (0–3). Confidence was established by questioning patients on their belief that the treatment received would actually help their back condition to improve. Finally, the baseline scores for the three outcome variables were considered for the purpose of stratification. It was hypothesized that those with lower initial scores (less disability and/or less subjective pain) may have less opportunity to and therefore be less likely to improve. The Roland-Morris scores were grouped as 0–6, 7–10, 11–14, and 15–24 (approximating quartiles). The pain variables were grouped as low (0–3), moderate (4–7) and high (8–10).

### Statistical methods

Binary logistic regression was used to estimate the effect of and test the association between BMI and the outcome variables, controlling for confounders. Odds ratios derived from the model results were used to measure effects. Comparisons were made between obese participants and non-obese participants for each of the three outcome variables.

Initially, binary logistic regression models included gender, race, age, treatment group, smoking status, alcohol consumption, coffee consumption, LBP history, duration of the current episode of LBP, baseline scores of the outcome variable, and patient confidence in the treatment. Gender, race, and smoking status were included because of a priori knowledge regarding their effects on LBP. Treatment group was also included to account for varying treatment protocols and their effects on treatment outcome.

Weight change, a history of LBP, and the duration of the current episode of LBP were considered as possibly having confounding influence on the BMI effect on treatment outcome. Likewise, patient confidence in treatment and the baseline scores of the outcome variable were also considered as possibly confounding the BMI effect. Models were then run with and without these variables to evaluate the influence each variable had on the effect of BMI on outcomes. Variables influencing the odds ratios of the remaining variables by more than 10 % were kept in the model. As a result, LBP history and weight change were eliminated from the disability and average pain models. LBP history was also removed from the severe pain model, but the weight change variable remained. Statistical analyses were performed using SAS (SAS Institute Inc., Cary, NC).

## Results

### Subject characteristics

There were few characteristics that differentiated obese from non-obese participants. Table 1 summarizes the distributions of sociodemographic, lifestyle and health-related characteristics. The distribution of obesity is also included as a percentage of the total number of participants in each respective category. Characteristics that appear to possibly differentiate participants by obesity include gender, race, and smoking status.

**Table 1:** Frequency and percentage of study patients by category of selected sociodemographic variables and by obesity within each category

Variable	Category	Number	Percent	Obese at baseline	Percent
Gender	Male	310	47.5	72	23.2
	Female	342	52.5	111	32.5
Age in Years	18–29	57	8.7	15	26.3
	30–39	142	21.8	41	28.9
	40–49	119	18.3	33	27.7
	50–59	122	18.7	35	28.7
	60–69	91	14.0	25	27.4
	70–79	95	14.6	29	30.5
	80–95	26	4.0	5	19.2
Race	White	396	60.7	84	21.2
	Non-White	256	39.3	99	38.7
Treatment Group	DC	165	25.3	36	21.8
	DC/Modalities	163	25.0	51	31.3
	MD	165	25.3	48	29.1
	MD/PT	159	24.4	48	30.2
Back Pain (Prior episode)	Yes	536	82.2	150	28.0
	No	116	17.8	33	28.4
Duration (this episode)	<3 months	275	42.2	76	27.6
	3 months +	377	57.8	107	28.4

There was also an association between the baseline Roland-Morris and baseline NRS with Roland-Morris improvement scores and NRS improvement scores, respectively. Those with lower baseline values exhibited a lesser tendency to show improvement based on the outcome variables. There was also a clear association between the baseline values and obesity. Obese participants tended to have higher baseline scores, and therefore had a greater opportunity for improvement. Baseline values were predictive of outcomes among non-obese subjects. Stratification on these baseline variables was warranted.

### Estimates of effects of obesity on improvement

Table 2 shows the adjusted effects of predictors on disability improvement (3+ points) as measured with the Roland-Morris questionnaire. Obese participants were less likely to show improvement following 6 months of treatment. BMI was not predictive within the overweight and underweight populations when compared to the normal weight (referent group) population.

**Table 2:** Estimated adjusted effects of BMI on 6-month improvement in disability as measured using Roland-Morris questionnaire (3+ points)

Category	Odds ratio	95 % confidence ratio
Underweight	1.05	(0.48, 2.30)
Normal weight	1.00^a^	-
Over weight	1.05	(0.67, 1.66)
Obese	0.62	(0.38, 1.00)

Adjusted for gender, race, treatment group, smoking status, duration of LBP, and confidence in treatment effect

^a^Reference category

Using average pain level as the outcome variable (2+ points, Table 3) produced some differences in results. BMI had no significant predictive value.

**Table 3:** Estimated Adjusted Effects of BMI on 6-Month Improvement (2+ points) in Subjective Average Back Pain

Category	Odds ratio	95 % confidence interval
Underweight	1.03	(0.48, 2.19)
Normal weight	1.00^a^	-
Over weight	0.95	(0.60, 1.48)
Obese	0.70	(0.44, 1.13)

Adjusted for gender, race, treatment group, smoking status, duration of LBP, and confidence in treatment effect

^a^Reference category

Finally, when modeling the patient’s most severe pain as the outcome variable (2+ points, Table 4), obesity once again appears to be predictive. The obese population was less likely to report an improvement in most severe pain scores. Overweight and underweight populations did not appear to significantly differ from the normal weight populations. Also, in this model weight gain was associated with a poorer outcome.

**Table 4:** Estimated Adjusted Effects of BMI on 6-Month Improvement (2+ points) in Subjective Most Severe Back Pain

Category	Odds ratio	95 % confidence interval
Underweight	0.70	(0.36, 1.65)
Normal weight	1.00^a^	-
Over weight	0.82	(0.52, 1.28)
Obese	0.54	(0.34, 0.88)

Adjusted for gender, race, treatment group, smoking status, duration of LBP, confidence in treatment effect, and weight gain

^a^Reference category

## Discussion

In an attempt to assess the soundness of recommending weight loss as a treatment for LBP, this study attempted to evaluate the role of BMI (and obesity) on participants’ responses to treatment for LBP. Previous studies have examined BMI as a risk factor for new onset LBP with mixed results [5]. Literature also indicates a weak association between obesity and the development of LBP may exist [22, 23]. However, the results of this study indicate that there may be an association between BMI and treatment outcome among participants randomized to medical or chiropractic care. While mean changes in low back pain intensity and disability were similar among the treatment groups [20], obesity may have an association with not having a positive outcome to treatment. This study also indicates that patients whose back pain is of a shorter duration are more likely to report improvement in their condition. Non-whites were also less likely to show improvement. These appear to be the only positive predictors of improved treatment outcomes within this study. Gender and treatment group (chiropractic or medical care with or without physical modalities) both failed to demonstrate any strong or consistent associations with the treatment outcomes.

The predictive value of weight gain on most severe pain scores (and not on disability and average pain scores) and smoking status on average pain scores may simply be chance findings.

Baseline disability and pain scores predicted outcomes. Clearly, those with higher baseline scores have more opportunity for improvement. However, disability improvement was considered a positive change of at least three and NRS improvement was considered a positive change of at least two. Within this framework, 93.6 % of participants had the opportunity to show improvement on disability (had baseline disability scores of three or higher). Likewise, 95.1 and 98.9 % had the opportunity to show improvement on the average and most severe pain scales, respectively (had baseline values of two or higher). It was considered that perhaps lower scores were indicative of a more chronic condition, less responsive to treatment. However, correlation procedures and contingency tables between duration of the episode and the improvement scores did not show any correlation. It is likely that many of the higher scores represented a temporary increase, perhaps severe enough to cause patients to seek care when they otherwise may not have, and spontaneous recovery may have occurred independent of treatment.

Results of this study suggest that BMI is a relevant predictor of response to treatment. Obese participants are less likely to show improvement from LBP treatment regardless of the care they receive.

The following limitations must be considered within the context of this study. Using BMI to indicate obesity may misclassify individuals who are excessively muscular and not excessively fat. Fat distribution must also be taken into account. Having excessive fat in the abdomen is considered riskier for developing LBP than when it is widespread through the body. Other measures of obesity involving skinfold measurements and body proportions may have yielded more accurate results. Non-obese patients with positive treatment outcomes included with the obese population would diminish the true effect of obesity on treatment outcomes.

There is potential for non-participation bias. The fact that 43.4 % of eligible participants did not participate may threaten the internal validity of the study. It is possible that obese participants who were less likely to improve were also less likely to participate, causing estimates to be biased due to selection. Participation may have been dependent on both prognosis and obesity. The minimal loss to follow-up in this study is a strength worth noting.

It is important to note that the data used in this study was originally collected 16–21 years ago. It is uncertain what effect, if any, this may have on the relevance of study outcomes on today’s population. However, there is no reason to believe that associations would be different in a more current LBP patient population.

Patient confidence may be weakly associated with an improved treatment outcome. Patients’ mental status should be considered when evaluating treatment outcomes, especially when self-reported subjective scales are used as with this study. Mental status could be a potential confounder of the obesity effect since depression may be predictive of obesity [24, 25]. Associations between back pain and measures of obesity may be stronger in individuals with an emotional disorder [26]. Additionally, since obesity may be associated with depression, mental status may be an intermediate in the causal pathway between BMI and treatment outcome. Data were collected on the subject’s mental status (SF-36) and the influence of these data were evaluated to assess the effect of psychological distress on low back pain as well as the effect of pain on subsequent distress [27].

## Conclusion

An association between obesity and less favorable treatment outcomes was inferred in this study. There appears to be an association (*p*-value ≤ 0.05) between obesity and disability as well as obesity and subjective most severe pain. Individuals who gained weight (5 or more pounds) were less likely to report improvement in subjective most severe pain. Therefore, it is reasonable to conclude that an association exists between obesity and the prognosis of treatment for LBP.

Future studies with a larger sample size are needed to determine if the findings of this study are maintained over a longer follow-up period.

## Abbreviations

BMI: Body mass index
HMO: Health maintenance organization
LBP: Low back pain
NRS: Numerical rating scale
